# Supplementary material for: MetaQTL: a package of new computational methods for the meta-analysis of QTL mapping experiments
Source: BMC Bioinformatics. 2007 Feb 8;8:49. doi: 10.1186/1471-2105-8-49 (PMC1808479; doi:10.1186/1471-2105-8-49)
Supplement: Additional File 7 — MetaQTL Package : jar file and tutorial. This Zip archive contains both the MetaQTL JAR file and the files of the tutorial. [file 1471-2105-8-49-S7.zip › org.inra.metaqtl/doc/org/metaqtl/bio/package-tree.html]

org.metaqtl.bio Class Hierarchy


|  |  |  |
| --- | --- | --- |
| **Overview** \| **Package** \| Class \| Use \| **Tree** \| **Deprecated** \| **Index** \| **Help** | |  |
| **PREV**   **NEXT** | **FRAMES**    **NO FRAMES**     **All Classes** |  |


---


## Hierarchy For Package org.metaqtl.bio

**Package Hierarchies:**: All Packages

---

## Interface Hierarchy

- org.metaqtl.bio.**IBioAdaptable**
  - org.metaqtl.bio.**IBioEntity**
    - org.metaqtl.bio.**IBioAllele**- org.metaqtl.bio.**IBioGenome**- org.metaqtl.bio.**IBioIndividual**- org.metaqtl.bio.**IBioLGroup**- org.metaqtl.bio.**IBioLocus**- org.metaqtl.bio.**IBioOntology**- org.metaqtl.bio.**IBioOntologyTerm**- org.metaqtl.bio.**IBioPopulation**
                    - org.metaqtl.bio.**IBioCross**
  - org.metaqtl.bio.**IBioAdapter**- org.metaqtl.bio.**IBioCrossTypes**- org.metaqtl.bio.**IBioEntityTypes**- org.metaqtl.bio.**IBioIndividualTree**- org.metaqtl.bio.**IOntologyTermProperties**

---


|  |  |  |
| --- | --- | --- |
| **Overview** \| **Package** \| Class \| Use \| **Tree** \| **Deprecated** \| **Index** \| **Help** | |  |
| **PREV**   **NEXT** | **FRAMES**    **NO FRAMES**     **All Classes** |  |


---
